# Changes in Sexual Risk Behavior in the Mombasa Cohort: 1993–2007

**DOI:** 10.1371/journal.pone.0113543

**Published:** 2014-11-21

**Authors:** Susan M. Graham, Janet Raboud, Walter Jaoko, Kishor Mandaliya, R. Scott McClelland, Ahmed M. Bayoumi

**Affiliations:** 1 Departments of Medicine and Global Health, University of Washington, Seattle, Washington, United States of America; 2 Department of Medical Microbiology, University of Nairobi, Nairobi, Kenya; 3 Dalla Lana School of Public Health, University of Toronto, Toronto, Ontario, Canada; 4 Toronto General Research Institute, University Health Network, Toronto, Ontario, Canada; 5 PathCare, Mombasa, Kenya; 6 Department of Epidemiology, University of Washington, Seattle, Washington, United States of America; 7 Centre for Research on Inner City Health, Keenan Research Centre of the Li Ka Shing Knowledge Institute and Division of General Internal Medicine, St. Michael's Hospital, Toronto, Ontario, Canada; 8 Department of Medicine and Institute of Health Policy, Management, and Evaluation, University of Toronto, Toronto, Ontario, Canada; Duke University Medical Center, United States of America

## Abstract

The Mombasa Cohort is an open cohort study following HIV-seronegative women reporting transactional sex. Established in 1993, the cohort provides regular HIV counseling and testing at monthly visits. Over time, HIV acquisition risk has declined steadily in this cohort. To evaluate whether this decline may reflect changes in sexual risk behavior, we investigated trends in condom use and partner numbers among women who participated in the Mombasa Cohort between 1993 and 2007. Multinomial logistic regression and generalized estimating equations were used to evaluate the association of calendar time and follow-up time with key risk behaviors, after adjustment for potential confounding factors. At enrollment visits by 1,844 women, the adjusted probability of never using condoms decreased over time, from 34.2% to 18.9%. Over 23,911 follow-up visits, the adjusted probabilities of reporting >2 partners decreased from 9.9% to 4.9% and inconsistent condom use decreased from 7.9% to 5.3% after ≥12 cohort visits. Important predictors of risk behavior were work venue, charging low fees for sex, and substance abuse. Women with a later sexual debut had less risky behavior. Although sexual risk has declined among women participating in the Mombasa Cohort, HIV acquisition continues to occur and interventions to promote and reinforce safer sex are clearly needed.

## Introduction

Female sex workers (FSW) have played an important role in the global HIV pandemic [1], particularly in sub-Saharan Africa [2]–[9]. Transactional sex work is widespread on the African continent, affecting not only urban centers but also rural communities, especially along migrant routes [5]. Recent evidence confirms that FSW remain at high risk for HIV throughout sub-Saharan Africa [1], [10]. For example, a systematic review and meta-analysis published in 2012 concluded that even in generalized epidemics, FSW have a greater than 12-fold increased odds of living with HIV as compared with women who are not sex workers [10]. A 2009 Kenyan Modes of Transmission study estimated that FSW and their clients accounted for approximately 14% of incident HIV infections [11].

Prospectively collected data on sexual risk behavior among women engaging in transactional sex are an important resource for the development of effective prevention interventions. The Mombasa Cohort, a prospective study of high-risk women in Mombasa, Kenya, is one of the longest continuously running high-risk cohorts in Africa and provides a unique opportunity to examine changes in risk behavior over time [12]. Participating women exchange sex for payment, in cash or in kind. Most (74%) work as barmaids, supplementing their income with occasional transactional sex work; others (21%) work in nightclubs, where they may have multiple partners or engage in serial monogamous relationships with paying clients.

HIV incidence has been monitored closely in this cohort throughout its existence. An analysis published in 2000 demonstrated a 10-fold decline in HIV-1 incidence over 3 years of follow-up, accompanied by significant declines in both sexually transmitted infection (STI) incidence and high-risk sexual behaviors during follow-up [13]. Between 1993 and 2007, 293 women had documented HIV-1 acquisition during follow-up, for an incidence of 6.4 per 100 person-years of observation (pyo) in this period (95% confidence interval [CI], 5.7 to 7.1 per 100 pyo). Incidence per 100 pyo declined steadily over the five 3-year periods covered, from 13.4 in 1993–1995 to 9.9 in 1996–1998 to 5.3 in 1999–2001 to 3.9 in 2002–2004, and finally to 2.4 in 2005–2007 (unpublished data). The purpose of this analysis was to evaluate changes in sexual risk behavior over this time period, in order to determine if there was evidence of risk reduction.

We hypothesized that behavior change may have occurred in the community over time, due to increasing HIV awareness and changing social norms, and that such changes would be reflected by differences in cohort enrollees over time. In addition, we hypothesized that ongoing counseling and provision of free condoms at cohort visits may have led to reduced levels of risky sexual activity among cohort participants. Finally, we hypothesized that risky behaviors would be associated with demographic, work venue, and health characteristics of the participants. Because an assessment of trends in risk behavior over time must account for changes in other characteristics of the population that may affect risk, we adjusted for several potentially confounding factors.

## Materials and Methods

### Population and Included Visits

All initially HIV-seronegative women who enrolled in the Mombasa Cohort between 1993 and 2007 were eligible for inclusion. Thus, both women who remained seronegative throughout follow-up and women who seroconverted during follow-up were included for analysis. Women were recruited using outreach meetings at night clubs, bars, guesthouses, and other sex work venues in the Mombasa area. Women who tested seronegative for HIV were eligible for enrollment if they were 18 years of age or older, willing to attend monthly follow-up visits with regular screening for HIV and STI, and self-identified as having exchanged sex for money or goods in the past month. The study used an open cohort design, with new enrollments on a continuing basis and no defined time limit to participation. Eligibility criteria did not change at any time during the 15-year follow-up period.

To exclude changes in risk behavior resulting from HIV diagnosis, we censored data for participants who acquired HIV during follow-up at the estimated date of infection (17 days prior to the first sample positive for HIV-1 RNA if detected prior to seroconversion, or mid-way between the last seronegative and the first seropositive visit) [14], [15]. We also excluded visits that occurred while participants were enrolled in two small randomized controlled trials (RCT), because the interventions tested (i.e., a nonoxynol-9 product and periodic presumptive treatment of vaginitis) may have influenced sexual risk behavior [16], [17]. All visits prior to RCT participation were included. RCT participants were allowed to rejoin the cohort after trial conclusion or were censored at RCT enrolment if they acquired HIV or were lost to follow-up during trial participation. We conducted two sensitivity analyses: one in which a marker was used to identify visits contributed by women after trial participation and the other in which all visits were included, with adjustment for RCT visit status. Because results were very similar to those of our main analyses, we do not present them in this manuscript.

### Clinic Procedures

Detailed study procedures have been described previously [15]. Briefly, data on demographic characteristics, sexual behavior, and medical history were collected at enrollment using a standardized questionnaire. Women then underwent a standardized physical examination, including pelvic speculum examination with screening for STI. At monthly follow-up visits, participants were asked about interim sexual behavior and health status before a repeat physical examination, STI screening, and HIV testing. Participants received risk-reduction counseling and free condoms at every visit. STI treatment and HIV care, including monitoring for disease progression and treatment of opportunistic infections, were provided free of charge. Antiretroviral therapy became available in 2004, after which treatment was offered to eligible HIV-infected women in accordance with Kenyan Ministry of Health guidelines.

### Ethics

All participants provided written informed consent. The cohort study was approved by the ethical review committees of the University of Nairobi and University of Washington. The analysis herein was also approved by the Office of Research Ethics at the University of Toronto.

### Risk Behavior Outcomes

At enrollment, women were asked to report the average weekly number of sex partners, sex acts, and sex acts with a condom over the past month. At follow-up visits, the period for recall was the past 7 days. A partner was defined as any sexual partner, whether regular or casual, paying or non-paying. Condom use was calculated by dividing the number of times the participant reported using a condom for sex by the number of times she reported having sex, multiplied by 100. The primary outcomes were reported number of sexual partners and percentage of reported coital acts protected by a condom. Reported partner numbers were categorized as more than two partners, two partners, one partner (reference category), and no partner. Reported condom use was categorized as never use (0% protected), inconsistent use (1%–99% protected), and always use (reference category).

### Enrollment Analysis

To analyze changes in risk behavior reported at enrollment, multinomial logistic regression was used to estimate associations between calendar time (categorized as 1993–1995, 1996–1998, 1999–2001, 2002–2004, and 2005–2007) and each partner number or condom use category, comparing each later year category to 1993–1995. Potential confounding factors identified a priori that may have changed over time among cohort participants included demographic (i.e., age, marital status, religion, education), sex work-related (i.e., venue, charge for sex, duration of sex work), and health (i.e., sexual debut, pregnancy, parity, hormonal contraceptive use, recent illness, substance use) characteristics. Fit for multivariable models was assessed using classification accuracy, the pseudo-R^2^ (Cox and Snell), and Akaike's information criterion (AIC) [18]–[20]. Based on these assessments, final multivariable models included year category and all predictors associated with outcomes at p<0.10 in a full model containing all a priori confounding factors, after checking for collinearity by calculating variance inflation factors and condition indices. Results are presented as relative-risk ratios (RRR) with 95% confidence intervals (CI) based on robust standard errors [21]–[23]. For example, an RRR of 0.60 for never using condoms in 2005–2007 means women were 40% less likely to report never versus always using condoms in this year category, relative to their reported use in 1993–1995. Adjusted probabilities were calculated using the method of recycled predictions [24]. Stata version 11.1 (StataCorp, College Station, TX, USA) was used for multinomial regression.

### Follow-Up Analysis

To analyze changes in risk behavior over follow-up visits, multinomial logistic regression with generalized estimating equations (GEE) was used to estimate associations between visit number (categorized as follow-up visits 1–2, 3–6, 7–12, and >12) and each partner number or condom use category, comparing each later category to visits 1–2. The GEE analysis used an exchangeable correlation matrix and robust variance estimation [25]. Multivariable analysis was conducted as for the enrollment analysis, with the same a priori confounding factors that may have changed over time among cohort participants included either as time-dependent (e.g., year category) or fixed variables (e.g., religion). Final multivariable models included visit category, year category, and all predictors associated with outcomes at p<0.10 in a full model containing all a priori confounding factors, after excluding collinearity. Results are presented as RRR with 95% CI based on robust standard errors [21]–[23]. SAS version 9.2 (SAS Institute Inc., Cary, NC, USA) and SAS-callable SUDAAN version 10.0.1 (RTI International, Research Triangle Park, NC) were used for analysis. Adjusted probabilities (i.e., mean predicted marginal probabilities) were calculated using post-estimation commands in SUDAAN. These probabilities are interpreted as the effect of visit category, holding other characteristics constant [25].

## Results

Between 1993 and 2007, 1,844 women enrolled in the cohort and met eligibility criteria (i.e., HIV-uninfected with non-RCT study visits). The distribution of enrollments over the 15-year period was 580 (31.4%) in 1993–1995, 544 (29.5%) in 1996–1998, 231 (12.5%) in 1999–2001, 254 (13.8%) in 2002–2004, and 235 (12.7%) in 2005–2007. Several potential confounding factors demonstrated trends over enrolment periods. For example, mean age at enrollment increased over time, from 27.3 years in the first period to 29.3 years in the last period. In addition, the proportion of enrollees who were night club workers decreased from 26.9% in the first period to 13.0% in the last, while the proportion who worked from home increased from 1.0% in the first period to 16.2% in the last.

After enrollment, 1,567 women (85.0%) contributed 18,063 follow-up visits, with a median interval between visits of 35 days (IQR, 28 days–55 days). Total follow-up time was 4,150 person-years of observation, with a median of 1.24 years per participant (IQR, 0.28 years–3.68 years) over a median of 5 visits (IQR, 2 visits–14 visits). The distribution of visits over the four visit categories was 2,836 (15.7%) at visits 1–2; 3,199 (17.7%) at visits 3–6; 2,835 (15.7%) at visits 7–12; and 9,193 (50.9%) at visits >12. Several potential confounding factors demonstrated trends over follow-up visit categories. For example, mean age increased over visits, from 27.8 at visits 1–2 to 34.9 at visits >12. In addition, the proportion of visits contributed by barmaids decreased from 72.8% at visits 1–2 to 63.5% at visits >12, while the proportion of visits contributed by night club workers increased from 22.4% at visits 1–2 to 33.8% at visits >12.

### Condom Use at Enrollment

**Figure 1:**
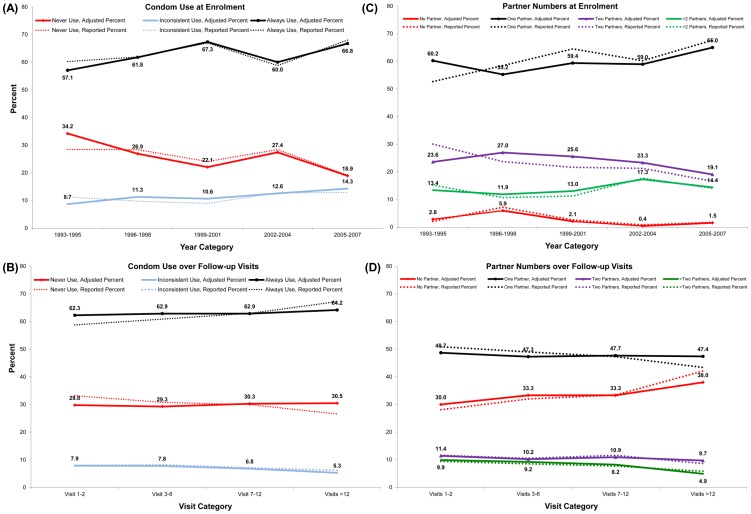
Trends in Condom Use and Partner Numbers in the Mombasa Cohort. Each panel presents the reported and adjusted percent of women reporting each outcome: (A) condom use at enrolment, (B) condom use over follow-up visits, (C) partner numbers at enrolment, and (D) partner numbers over follow-up visits. Solid lines represent adjusted percentages derived from modeling, with numeric values indicated in bold. Dotted lines represent the percent of women reporting each behavior in the time period or visit category indicated.

Among 1,769 women (95.9% of enrollees) who reported that they were sexually active during the month prior to enrollment, 472 women (26.7%) reported never using condoms, 194 women (11.0%) used condoms inconsistently, and 1,103 women (62.4%) always used condoms. Among women reporting inconsistent condom use, the proportion of sex acts with condoms ranged from 5.6% to 83.3%. The percentage of women reporting never using condoms decreased from 28.4% in 1993–1995 to 19.1% in 2005–2007; the percentage of women reporting always using condoms increased from 60.2% to 68.0% over the same period. Inconsistent condom use ranged from 11.3% in 1993–1995 to 12.9% in 2005–2007. Figure 1A presents the unadjusted trends over enrollment periods (dashed lines).

After adjustment for potential confounding factors, there was a significant decrease in never using condoms (relative to always using condoms) in all time periods (relative to 1993–1995) except for 2002–2004 (Table 1). In contrast, there was no change in reporting inconsistent condom use relative to always using condoms. Adjustment for potentially confounding factors accentuated the differences in condom use trends over time (Figure 1A, bold lines).

**Table 1:** Trends in Reported Condom Use at Enrollment, Multivariable Analysis^a^^,^ b.

		*Never Use*			*Inconsistent Use*		
*Predictor*		*aRRR*	*95% CI*	*P value*	*aRRR*	*95% CI*	*P value*
Year category	1993–1995	Reference			Reference		
	1996–1998	0.66	0.47–0.93	0.02	1.22	0.79–1.88	0.37
	1999–2001	0.47	0.31–0.71	<0.001	1.05	0.58–1.88	0.88
	2002–2004	0.70	0.47–1.04	0.08	1.41	0.83–2.37	0.20
	2005–2007	0.39	0.25–0.62	<0.001	1.46	0.87–2.44	0.15
Workplace	Bar or guesthouse	Reference			Reference		
	Nightclub	0.20	0.10–0.39	<0.001	0.62	0.35–1.07	0.08
	Home-based or other	0.62	0.31–1.24	0.17	0.75	0.34–1.66	0.48
Charge category^c^	Non-monetary exchange	Reference			Reference		
	Low charge	0.26	0.20–0.34	<0.001	2.60	1.70–3.96	<0.001
	Medium charge	0.17	0.09–0.31	<0.001	1.71	0.90–3.24	0.10
	High charge	0.09	0.04–0.22	<0.001	0.75	0.33–1.67	0.48
Duration of sex work	≤1 year	Reference			Reference		
	>1-<4 years	1.64	1.20–2.24	0.002	1.61	1.07–2.40	0.02
	≥4 years	1.31	0.94–1.82	0.11	1.83	1.23–2.72	0.003
Sexual debut	≤15 years	Reference			Reference		
	16–17 years	0.85	0.62–1.19	0.35	1.06	0.71–1.57	0.78
	≥18 years	0.91	0.67–1.24	0.55	0.62	0.42–0.93	0.02
Pregnancy	Pregnant at enrollment	2.47	1.26–4.84	0.009	2.24	0.92–5.46	0.08
Recent illness	Ill in past month	1.00	0.57–1.76	0.99	1.92	1.10–3.36	0.02
Alcohol use	Drinks alcohol	0.65	0.49–0.87	0.003	1.66	1.07–2.59	0.02
Religion	Muslim	0.63	0.38–1.03	0.07	0.69	0.40–1.19	0.18

aRRR  =  adjusted relative-risk ratio.

^a^Reference category is: Always Use.

^b^P value for year category in model is <0.001.

^c^Charge for sex was divided into four categories: non-monetary, low charge (1–500 Kenyan shillings), medium charge (501–1000 Kenyan shillings), high charge (>1000 Kenyan shillings), based on knowledge of the population and work venues. One US dollar equals approximately 87 Kenyan shillings.

We also examined the relationship between condom use categories and other characteristics (Table 1). Never using condoms was much less common than always using condoms among women working in night clubs and charging cash for sex. In addition, never using condoms had a strong positive association with being pregnant at enrollment. Inconsistent condom use was less common than always using condoms among women with a later sexual debut. Inconsistent condom use had positive associations with charging the lowest fee for sex, having a longer duration of sex work, reporting a recent illness, and reporting alcohol use.

### Partner Numbers at Enrollment

The average number of partners per week reported at enrollment was zero for 63 women (3.4%), one for 1,084 women (58.8%), two for 446 women (24.2%), and more than two for 250 women (13.6%). The percentage of women reporting one partner per week increased over time, from 52.7% in 1993–1995 to 67.7% in 2005–2007, while the percentage of women reporting two partners decreased from 30.1% to 16.6% over the same period. The percentage of women reporting more than two partners per week ranged from 10.7% to 17.7%. The percentage of women reporting no partner at enrolment increased from 2.1% in 1993–1995 to 7.2% in 1996–1998, after which this group remained small (<3.0% total). Figure 1C presents the unadjusted trends over enrollment periods (dashed lines).

After adjustment for potential confounding factors, there was no change over time in the percentage of women reporting two or more partners (relative to one partner, Table 2). An increase in reporting no partner (relative to one partner) was observed in 1996–1998 only. Adjustment for potentially confounding factors attenuated changes in partner numbers over time (Figure 1C, bold lines).

**Table 2:** Trends in Reported Partner Numbers at Enrollment, Multivariable Analysis^a^^,^ b.

		*No Partner*			*Two Partners*			*More Than Two Partners*		
*Predictor*		*aRRR*	*95% CI*	*P value*	*aRRR*	*95% CI*	*P value*	*aRRR*	*95% CI*	*P value*
Year category	1993–1995	Reference			Reference			Reference		
	1996–1998	2.39	1.12–5.14	0.02	1.24	0.88–1.74	0.23	0.95	0.60–1.50	0.82
	1999–2001	0.73	0.25–2.13	0.56	1.13	0.74–1.74	0.57	1.03	0.55–1.92	0.93
	2002–2004	0.14	0.02–1.15	0.07	1.13	0.74–1.72	0.56	1.63	0.93–2.85	0.09
	2005–2007	0.52	0.16–1.65	0.26	0.72	0.44–1.16	0.18	0.95	0.48–1.87	0.88
Workplace	Bar or guesthouse	Reference			Reference			Reference		
	Nightclub	1.32	0.28–6.10	0.72	2.94	1.99–4.36	<0.001	12.37	7.25–21.09	<0.001
	Home-based or other	1.16	0.15–9.30	0.89	2.19	1.10–4.39	0.03	11.67	5.65–24.13	<0.001
Charge category^c^	Non-monetary exchange	Reference			Reference			Reference		
	Low charge	0.11	0.03–0.38	<0.001	8.14	5.80–11.42	<0.001	29.51	12.32–70.69	<0.001
	Medium charge	.	.	.	7.13	4.39–11.58	<0.001	19.25	7.46–49.66	<0.001
	High charge	0.17	0.01–1.93	0.15	7.24	4.33–12.11	<0.001	38.07	14.77–98.15	<0.001
Duration of sex work	≤1 year	Reference			Reference			Reference		
	>1–<4 years	0.74	0.35–1.58	0.44	1.46	1.05–2.02	0.02	1.63	1.05–2.54	0.03
	≥4 years	0.48	0.19–1.20	0.12	1.58	1.12–2.23	0.009	1.58	1.00–2.48	0.05
Sexual debut	≤15 years	Reference			Reference			Reference		
	16–17 years	0.98	0.49–1.96	0.96	1.08	0.79–1.49	0.62	1.18	0.75–1.84	0.47
	≥18 years	0.77	0.39–1.50	0.44	0.63	0.46–0.86	0.004	0.75	0.49–1.15	0.19
Parity	Ever live birth	0.86	0.39–1.88	0.70	0.68	0.49–0.94	0.02	0.70	0.46–1.06	0.09
Alcohol use	Drinks alcohol	0.47	0.26–0.86	0.01	1.83	1.32–2.54	<0.001	2.36	1.50–3.69	<0.001
Illicit drug use	Uses illicit drugs	.	.	.	3.65	1.07–12.46	0.04	4.22	1.06–16.82	0.04
Marital status	Ever married	2.74	1.45–5.17	0.002	1.32	0.99–1.75	0.06	1.37	0.93–2.02	0.11

aRRR  =  adjusted relative-risk ratio.

^a^Reference category is: One Partner.

^b^P value for year category in model is 0.003.

^c^Charge for sex was divided into four categories: non-monetary, low charge (1–500 Kenyan shillings), medium charge (501–1000 Kenyan shillings), high charge (>1000 Kenyan shillings), based on knowledge of the population and work venues. One US dollar equals approximately 87 Kenyan shillings.

We also examined the relationship between partner numbers and other characteristics (Table 2). Reporting no partner was strongly negatively associated with using alcohol and charging the lowest fee for sex, and positively correlated with previous marriage. Reporting two or more partners was most strongly positively associated with working in night clubs or other venues, charging cash payments for sex, drinking alcohol, and using illicit drugs.

### Condom Use over Follow-Up

Data on condom use in the last week were available for 11,395 of 11,413 visits (99.8%) on which women reported having been sexually active. Overall, never use of condoms was reported at 29.1%, inconsistent use at 7.0%, and always use at 63.8% of visits. At visits where condom use was inconsistent, the reported proportion of sex acts with a condom ranged from 8.7% to 95.0%. Visits on which women reported no sexual activity (6,637 or 36.7%) and visits on which data on sexual behavior were missing (13 or 0.1%) were excluded from the analysis of condom use over follow-up. Comparing reports after 1–2 visits to after 12 or more visits, never use of condoms decreased from 33.2% to 26.6%, inconsistent use decreased slightly from 8.0% to 6.2%, and always using condoms increased from 58.8% to 67.2%. Figure 1B presents the unadjusted trends over follow-up periods (dashed lines).

After adjustment for potential confounding factors, the probability of never using condoms relative to always using condoms did not change over visits (Table 3). Reporting inconsistent condom use was less likely among women attending 12 or more visits relative to those attending the first two visits. Trends in condom use over follow-up were greatly attenuated after adjustment for potential confounding factors and intra-individual correlation (Figure 1B, bold lines).

**Table 3:** Trends in Reported Condom Use over Follow-up, Multivariable Analysis^a^^,^ b.

		*Never Use*			*Inconsistent Use*		
*Predictor*		*aRRR*	*95% CI*	*P value*	*aRRR*	*95% CI*	*P value*
Visit category^c^	Visits 1–2	Reference			Reference		
	Visits 3–6	0.97	0.84–1.11	0.63	0.98	0.77–1.24	0.86
	Visits 7–12	1.01	0.85–1.19	0.93	0.84	0.63–1.14	0.27
	Visits >12	0.99	0.82–1.20	0.93	0.65	0.47–0.89	0.008
Year category^c^	1993–1995	Reference			Reference		
	1996–1998	0.87	0.67–1.13	0.30	0.83	0.54–1.28	0.39
	1999–2001	0.73	0.55–0.97	0.03	0.64	0.39–1.05	0.08
	2002–2004	0.70	0.52–0.94	0.02	0.58	0.37–0.91	0.02
	2005–2007	0.83	0.59–1.15	0.26	0.94	0.62–1.42	0.77
Age category^c^	<23 years	Reference			Reference		
	23–26 years	1.05	0.81–1.37	0.70	1.45	0.96–2.20	0.08
	27–30 years	1.20	0.90–1.59	0.21	1.58	1.02–2.45	0.04
	>30 years	1.04	0.77–1.42	0.79	1.41	0.84–2.36	0.19
Workplace	Bar or guesthouse	Reference			Reference		
	Nightclub	0.25	0.17–0.37	<0.001	0.83	0.57–1.22	0.35
	Home-based or other	0.41	0.25–0.68	<0.001	1.05	0.59–1.85	0.87
Charge category^d^	Non-monetary exchange	Reference			Reference		
	Low charge	0.66	0.54–0.82	<0.001	1.36	0.98–1.87	0.06
	Medium charge	0.36	0.23–0.55	<0.001	0.80	0.49–1.32	0.39
	High charge	0.38	0.24–0.59	<0.001	0.83	0.51–1.35	0.45
Sex work before enrollment	≤1 year	Reference			Reference		
	>1-<4 years	1.42	1.12–1.80	0.004	1.10	0.81–1.51	0.54
	≥4 years	1.38	1.07–1.79	0.01	1.04	0.72–1.52	0.82
Sexual debut	≤15 years	Reference			Reference		
	16–17 years	0.83	0.65–1.05	0.12	1.04	0.74–1.44	0.84
	≥18 years	1.02	0.81–1.28	0.86	0.83	0.61–1.15	0.26
Pregnancy^c^	Pregnant at visit	2.12	1.57–2.87	<0.001	0.92	0.47–1.77	0.79
Recent illness^c^	Ill since last visit	1.21	1.05–1.41	0.01	0.79	0.57–1.08	0.13
Alcohol use	Drinks alcohol	1.10	0.88–1.39	0.40	1.47	1.07–2.02	0.02
Religion	Muslim	0.88	0.64–1.20	0.42	0.85	0.58–1.27	0.44

aRRR  =  adjusted relative-risk ratio.

^a^Reference category is: Always Use.

^b^P value for visit category in model is 0.20.

^c^Time-dependent covariate.

^d^Charge for sex was divided into four categories: non-monetary, low charge (1–500 Kenyan shillings), medium charge (501–1000 Kenyan shillings), high charge (>1000 Kenyan shillings), based on knowledge of the population and work venues. One US dollar equals approximately 87 Kenyan shillings.

We also examined the relationship between condom use and other characteristics (Table 3). Never using condoms strongly negatively correlated with working in night clubs or from home and charging cash for sex. Never using condoms was positively correlated with longer duration of sex work, recent illness, and pregnancy. Inconsistent condom use was positively correlated with alcohol use.

### Partner Numbers over Follow-Up

Data on partner numbers in the last week were available for 18,050 of 18,063 follow-up visits (99.9%). Overall, no sexual activity (i.e., no partner) was reported at 36.8%, one partner at 46.2%, two partners at 9.9%, and more than two partners at 7.2% of visits. Among women with more than two partners, the reported number of partners ranged from 3 to 28 partners in the past week. Visits on which data on partner numbers were missing (13 or 0.1%) are excluded from analysis. Comparing reports after 1–2 visits to after 12 or more visits, the percentage of women reporting no partner increased over visits from 28.1% to 42.1%, reports of one partner decreased from 50.9% to 43.4%, reports of two partners decreased from 11.6% to 8.6%, and reports of more than two partners decreased from 9.4% to 5.8%. Figure 1D presents the unadjusted trends over follow-up periods (dashed lines).

After adjustment for potential confounders, the probability of reporting no partner increased after the first two visits (Table 4). While the probability of reporting two partners did not change significantly over cohort visits, reporting more than two partners was less likely after 12 visits in the cohort. Apparent differences were attenuated by adjustment for intra-individual correlation and cohort make-up (Figure 1D, bold lines).

**Table 4:** Trends in Reported Partner Numbers over Follow-up, Multivariable Analysis^a^^,^ b.

		*No Partner*			*Two Partners*			*More Than Two Partners*		
*Predictor*		*aRRR*	*95% CI*	*P value*	*aRRR*	*95% CI*	*P value*	*aRRR*	*95% CI*	*P value*
Visit category^c^	Visits 1–2	Reference			Reference			Reference		
	Visits 3–6	1.15	1.02–1.29	0.02	0.91	0.76–1.09	0.30	0.93	0.76–1.14	0.48
	Visits 7–12	1.14	0.99–1.31	0.07	0.96	0.79–1.17	0.67	0.81	0.65–1.03	0.08
	Visits >12	1.32	1.14–1.52	<0.001	0.83	0.66–1.03	0.09	0.46	0.34–0.62	<0.001
Year category^c^	1993–1995	Reference			Reference			Reference		
	1996–1998	1.02	0.86–1.20	0.83	0.96	0.77–1.20	0.72	1.25	0.93–1.68	0.13
	1999–2001	1.34	1.09–1.65	0.005	0.74	0.56–0.99	0.04	1.16	0.79–1.71	0.44
	2002–2004	1.28	1.01–1.62	0.04	0.71	0.52–0.99	0.04	1.25	0.81–1.93	0.32
	2005–2007	0.96	0.74–1.24	0.73	0.70	0.49–1.01	0.06	1.58	0.99–2.52	0.06
Age category^c^	<23 years	Reference			Reference			Reference		
	23–26 years	1.02	0.83–1.24	0.88	1.08	0.86–1.36	0.49	0.78	0.57–1.08	0.13
	27–30 years	1.17	0.91–1.50	0.23	1.12	0.83–1.50	0.47	0.76	0.51–1.12	0.17
	>30 years	1.43	1.10–1.87	0.008	1.11	0.78–1.58	0.57	0.62	0.38–1.00	0.05
Workplace	Bar or guesthouse	Reference			Reference			Reference		
	Nightclub	1.10	0.88–1.37	0.40	2.39	1.81–3.15	<0.001	4.46	2.84–7.01	<0.001
	Home-based or other	0.93	0.61–1.42	0.73	3.81	2.28–6.34	<0.001	15.47	8.74–27.38	<0.001
Charge category^d^	Non-monetary exchange	Reference			Reference			Reference		
	Low charge	0.86	0.73–1.02	0.08	1.97	1.53–2.53	<0.001	6.32	4.07–9.80	<0.001
	Medium charge	1.20	0.90–1.60	0.20	2.06	1.45–2.92	<0.001	6.62	3.58–12.23	<0.001
	High charge	1.21	0.93–1.58	0.15	2.07	1.44–2.97	<0.001	7.14	3.95–12.89	<0.001
Sex work before enrollment	≤1 year	Reference			Reference			Reference		
	>1–<4 years	1.01	0.85–1.19	0.95	1.19	0.94–1.51	0.15	0.91	0.63–1.32	0.62
	≥4 years	1.25	1.04–1.51	0.02	0.84	0.64–1.11	0.22	1.00	0.66–1.50	0.98
Sexual debut	≤15 years	Reference			Reference			Reference		
	16–17 years	1.00	0.83–1.20	1.00	1.12	0.88–1.43	0.35	1.08	0.74–1.57	0.69
	≥18 years	1.04	0.88–1.23	0.67	0.82	0.65–1.04	0.11	1.03	0.72–1.46	0.88
Parity	Ever live birth	1.22	1.00–1.49	0.05	0.84	0.67–1.04	0.11	0.82	0.58–1.15	0.25
Alcohol use	Drinks alcohol	0.80	0.67–0.94	0.008	1.22	0.95–1.56	0.12	1.19	0.83–1.72	0.35
Illicit drug use	Uses illicit drugs	0.85	0.54–1.33	0.48	0.72	0.41–1.27	0.25	1.21	0.65–2.25	0.55
Marital status	Ever married	0.83	0.71–0.96	0.02	1.14	0.92–1.42	0.22	1.55	1.11–2.16	0.01

aRRR  =  adjusted relative-risk ratio.

^a^Reference category is: One Partner.

^b^P value for visit category in model is <0.001.

^c^Time-dependent covariate.

^d^Charge for sex was divided into four categories: non-monetary, low charge (1–500 Kenyan shillings), medium charge (501–1000 Kenyan shillings), high charge (>1000 Kenyan shillings), based on knowledge of the population and work venues. One US dollar equals approximately 87 Kenyan shillings.

We also examined the relationship between partner number categories and other characteristics (Table 4). Again, reporting no partner was negatively associated with using alcohol. Women over 30 years of age were more likely to report no partner. Reporting two or more partners was most strongly positively associated with working in night clubs or other venues and charging cash payments for sex.

## Discussion

Condom use reported at enrolment visits increased significantly among FSW recruited to this prospective cohort over the 15-year study period. In our adjusted analysis, the proportion of women reporting never using condoms decreased in each 3-year period except one since the cohort was initiated. During extended follow-up, there was a small but significant decrease in inconsistent condom use. However, only 67% of sexually active FSW in follow-up during the most recent period (2005–2007) reported always using a condom, despite regular risk reduction counseling and provision of free condoms.

Workplace was a very strong predictor of condom use, which was less frequent among women working at bars or guesthouses. This finding is consistent with a study of female sex workers in Nairobi [26]. It is unclear whether access to condoms or prevention information is problematic in this setting, or whether bar clients are particularly averse to condom use. Of note, condom use was more likely with cash transactions, which may be more common in night club settings. Inconsistent condom use was associated with alcohol use, as in other African studies [27], [28]. In addition, women charging very low cash fees for sex were more likely to use condoms inconsistently, in accordance with qualitative research in the Mombasa Cohort that identified economic pressure as an important barrier to safer sex in this population [29]. Women were also more likely to report inconsistent condom use after a recent illness, perhaps due to a temporary reduction in motivation or ability to obtain condoms. Finally, women with a later sexual debut were more likely to use condoms consistently, suggesting that these women may be more capable of negotiating safer sex.

The majority of women (59%) reported having had one partner in an average week over the month before enrollment, with 38% reporting two or more partners, and just 3% reporting no partner. Partner numbers reported at enrollment did not change greatly over time. Over cohort follow-up, more women (37%) reported no partner, and fewer (17%) reported two or more partners in the past working week. These results may reflect a difference in study methods: women were asked to report their weekly average number of partners over the past month at enrollment and subsequently were asked to report the number of partners in the past week only. The follow-up analysis was limited to visits using the same recall period and methodology, and identified a small decrease in the adjusted probability of reporting more than two partners during extended follow-up.

The most consistent associations of predictor variables with partner numbers across models were workplace, charge for sex, and alcohol use. The same workplace factors that were associated with consistent condom use (i.e., night club venue, cash payments for sex) were also associated with multiple sex partners. Women who charged the lowest fee for sex were less likely than others to report no partner in the past working week, likely reflecting a greater economic pressure on this group [29]. Women with a later sexual debut again reported less risky behavior, with a lower probability of reporting multiple partners. Given current interest in structural interventions to keep young girls in school and delay sexual activity [30], [31], it is intriguing that a later sexual debut was associated with less risky sexual behavior (i.e., more consistent condom use, fewer partners) in this population of adult women engaging in transactional sex.

Strengths of this study include the large number of participants and person-years of follow-up, with consistent procedures for data collection over the 15-year period. In addition, eligibility for cohort enrollment was based only on a recent history of having exchanged money or goods for sex, and women were allowed to participate for as long as they wished, regardless of ongoing risk behaviors. This inclusivity has ensured a diverse population of participating women, avoiding the over-reporting of high-risk behavior in order to gain or retain access to cohort services. Finally, this study overcomes some methodologic limitations of prior work on trends in sexual risk among women engaging in transactional sex. For example, a previous study also carried out in the Mombasa area reported large increases in both partner numbers and condom use in an unadjusted analysis comparing two cross-sectional surveys conducted in 2000 and 2005 [31]. However, no firm conclusions could be drawn about behavior trends, because the two surveys enrolled very different study populations [32]. The methods used in the current analysis permitted a flexible approach, including separate trend analyses for enrollment and follow-up data, the use of year and visit categories allowing for non-linear trends, the use of multinomial regression to assess differences between discrete categories of reported condom use and partner numbers, and adjustment for intra-individual correlation using GEE. This approach is likely to yield more accurate information on trends in sexual risk behavior.

This study had several limitations. First, all studies that use self-reported behavior are limited by recall and social desirability bias. When biomarkers of sexual activity have been used as a gold standard, women have been found to underreport their risk behavior [33]. Some misclassification bias was therefore likely in this study, although there is no reason to believe that such bias changed over calendar time or cohort visits. Second, no data were collected on the type and duration of partnerships. Therefore, no conclusion about differences in condom use with different partner types (e.g., regular partners versus clients) could be drawn. Several studies of FSW in East Africa have reported less frequent use of condoms with regular partners than clients [34]–[36]. Women in the Mombasa Cohort report that negotiating condom use is more difficult in long-term partnerships of any type [29]. Women with multiple partners who did not use condoms with one or more partner they consider “regular” would be classified as having inconsistent condom use in this analysis, despite consistent condom use with non-regular partners or clients. Third, this was an open cohort, with continuous enrollment of women who participated for varying lengths of time. As such, differential loss to follow-up between women with varying levels of behavioral risk may influence the results of this study. This effect is partially accounted for by adjusting the analysis for cohort make-up and intra-individual correlation using GEE. However, confounding due to unmeasured differences over time or visits may still bias these results. Finally, the women participating in the Mombasa Cohort volunteer for research visits with regular HIV and STI screening; as such, our findings may not be generalizable to all women engaging in transactional sex.

## Conclusions

In conclusion, this analysis demonstrated small but significant decreases in high-risk behavior among women engaging in transactional sex who enrolled and participated in the Mombasa Cohort over a 15-year period. The proportion of women reporting never using condoms at enrollment decreased over time, although partner numbers remained relatively stable. During extended follow-up, the rate of inconsistent condom use and the frequency of reporting more than two partners both decreased. However, approximately one third of women still reported never or inconsistent use of condoms at visits in the final 3-year period assessed, and reported partner numbers did not change dramatically over time. We identified a particular need for interventions targeting bars and guesthouses, where condom use was infrequent. In addition, interventions to reduce alcohol use and illicit drug use, address economic hardship (e.g., among women charging low fees for sex), and delay initiation of sexual activity may reduce sexual risk. Novel interventions targeting these problems are needed. While the HIV prevention education, counseling, and free condoms provided by this cohort study may have led to some risk reduction over time, HIV acquisition continues to occur and interventions to promote and reinforce safer sex are clearly needed.
